# MMTV Virus Detection, Survival Analysis, and Prognostic Relevance of Six Tumor Genes in Patients With Breast Cancer

**DOI:** 10.1155/ijbc/3310843

**Published:** 2026-04-12

**Authors:** Saad Alamri, Maaweya Awadalla, Rahaf A. Henawi, Ghaida Al-hazzaa, Zahra Alkhunaizy, Soha Alzorgi, Nouf Alqahtani, Alyaa S. Abdel Halim, Mohamed A. M. Ali, Mansour I. Almansour, Bandar Alosaimi

**Affiliations:** ^1^ Research Center, King Fahad Medical City, Riyadh Second Health Cluster, Riyadh, Saudi Arabia, kfmc.med.sa; ^2^ Pathology and Clinical Laboratory Medicine Administration, King Fahad Medical City, Riyadh Second Health Cluster, Riyadh, Saudi Arabia, kfmc.med.sa; ^3^ Anesthesiology and Operating Room Administration, King Fahad Medical City, Riyadh Second Health Cluster, Riyadh, Saudi Arabia, kfmc.med.sa; ^4^ Comprehensive Cancer Center, King Fahad Medical City, Riyadh Second Health Cluster, Riyadh, Saudi Arabia, kfmc.med.sa; ^5^ Department of Biochemistry, Faculty of Science, Ain Shams University, Cairo, Egypt, asu.edu.eg; ^6^ Department of Biology, College of Science, Imam Mohammad Ibn Saud Islamic University (IMSIU), Riyadh, Saudi Arabia, imamu.edu.sa; ^7^ Department of Zoology, College of Science, King Saud University, Riyadh, Saudi Arabia, ksu.edu.sa

**Keywords:** breast cancer, DNA repair, Kaplan–Meier survival analysis, mouse mammary tumor virus (MMTV), telomere length, tumor suppressor genes

## Abstract

**Background/Objectives:**

Breast cancer (BC) remains the most frequently diagnosed malignancy among women worldwide and represents a significant global health burden. The genes associated with tumor suppression (p53, BRCA1, and BRCA2), telomere length maintenance (TERT), DNA damage response (FGFR2), and DNA repair (CHD1) are recognized for their intricate function in tumor genesis and progression. However, the prognostic significance of these genes in BC remains an area of research interest. This study aimed to examine the potential association between the presence of the MMTV and the expression patterns of these six genes with patient prognosis and survival outcomes in BC.

**Methods:**

This study includes 125 formalin‐fixed, paraffin‐embedded (FFPE) tissue specimens taken from BC patients, in addition to 25 tissue samples of benign breast lesions were incorporated as controls. The mRNA expression levels of six genes, namely p53, BRCA1, BRCA2, TERT, FGFR2, and CHD1, were quantified in FFPE tissue samples using quantitative polymerase chain reaction (qPCR). The correlation between gene expression and prognostic characteristics and the probability of recurrence‐free survival (RFS) and overall survival (OS) were assessed.

**Results:**

The results do not indicate an association between MMTV and BC, as the virus was not detected in any of the tissue samples analyzed. We observed a significant differential expression in five of the six studied genes between BC and noncancerous breast tissue, with significant downregulation of BRCA1, BRCA2, CHD1, and TERT, significant upregulation of p53, and unchanged levels of FGFR2. Among BC patients, p53 and BRCA1 expression levels emerged as significant prognostic factors for both RFS (32 vs. 24 months; 34 vs. 26 months) and OS (28.5 vs. 24 months; 31 vs. 28 months), respectively. Kaplan–Meier survival analysis of p53 expression displayed a trend favoring low expression for better survival and showing relatively stable RFS and OS survival curves of p53 until 43 and 54 months of the follow‐up period, respectively.

**Conclusions:**

When comparing cancer to noncancer patients, only p53 and BRCA1 expression levels emerged as significant prognostic factors for both RFS and OS in the entire cohort, with p53 displaying a trend favoring low expression for better survival. Although gene expression data provided a prognostic value, future studies should aim at integrating multiomics data and evaluating biomarkers in a broader clinical context to improve the accuracy of prognostic models and guide personalized treatment strategies.

## 1. Introduction

Breast cancer (BC) remains the most frequently diagnosed malignancy among women worldwide and poses a significant global health burden. BC was the second most often diagnosed cancer in 2022, with around 2.3 million new cases globally, accounting for 11.6% of all malignancies worldwide. BC also ranks as the fourth leading cause of cancer‐related mortality, with 665,684 fatalities anticipated globally in 2022, constituting 6.9% of all cancer deaths worldwide, creating a significant public health issue [1].

The etiological heterogeneity of BC can be attributed to both hereditary and nongenetic risk factors. The majority of BC cases are sporadic, while inherited factors constitute 5%–10% of cases. Genetically, approximately 30 genes are identified as risk factors for BC. The genes encompass predisposed genes; high, moderate, and low penetrance genes; syndrome‐associated genes; single nucleotide polymorphisms; and common variants, which are likely to be mutated and serve as precursors for BC [2]. Nongenetic factors encompass age, reproductive risk factors (such as early menarche and late menopause), lifestyle factors (including obesity, smoking, and alcohol consumption), elevated mammographic density, oral contraceptive use, radiation exposure, and environmental pollutants, although certain factors may also be influenced by genetic predisposition. The potential role of mouse mammary tumor virus (MMTV) in human BC has been a subject of ongoing investigation. MMTV is a retrovirus known to induce mammary tumors in mice, and studies have suggested that it may also play a role in the etiology of some BCs in humans, particularly in a subset of BC cases exhibiting viral sequences in their genomes [3]. Viral, environmental, genetic, and hormonal factors compromise DNA integrity, resulting in both genetic and epigenetic alterations. These alterations facilitate cellular dysregulation and provoke abnormal cell progression, resulting in BC [4].

Early identification of BC is crucial for improving survival outcomes and reducing mortality rates. Consequently, several countries have implemented population‐based mammographic screening initiatives to facilitate earlier detection and reduce tumor burden, thereby enhancing survival rates [5]. Notwithstanding advancements in screening protocols and perioperative systemic medication, 25%–30% of individuals with early‐stage BC will ultimately progress to metastatic disease [6]. Despite significant advancements in survival rates over the last 20 years, the global incidence of BC persists in its upward trajectory, highlighting the necessity for enhanced prevention initiatives, earlier detection methods, tumor stratification, and optimal therapeutic approaches [7]. Due to its varied nature, traditional pathological diagnosis and histological classification are insufficient for the precise management of BC. Fortunately, modern understanding of BC has advanced to the molecular level, and the integration of classical morphological assessment with molecular profiling has facilitated enhanced treatment outcomes and improved care [8].

In the past decade, genetic testing utilizing gene expression patterns and mutations has significantly enhanced the accurate diagnosis and treatment of BC, facilitating the selection of adjuvant therapy and prognostic predictions. Methods that detect critical molecules in the development and evolution of BC through genetic testing can act as prognostic indicators and assist in customizing treatment methods to enhance patient outcomes [9]. More recently, the list of genes implicated in BC development and progression has expanded, indicating diverse implications from prognostic, preventative, and therapeutic perspectives. Identifying the genes that predict BC prognosis and identifying their functional mechanisms would significantly improve understanding of the etiology and progression of BC, thereby facilitating personalized treatment, promoting the advancement of personalized medicine, and enhancing BC treatment and prognosis. Recent studies have shown the efficacy of gene expression signatures in identifying critical genes linked to the prognosis of BC [10].

The genome of breast tumor cells is deemed unstable, evidenced by numerous chromosomal and genetic abnormalities. Several molecular markers play significant roles in BC susceptibility and progression. TP53 (Tumor Protein p53) is frequently mutated in BC and is associated with poor prognosis due to its central role in regulating cell cycle arrest, DNA repair, and apoptosis [11]. BRCA1 and BRCA2 are tumor suppressor genes crucial for homologous recombination DNA repair; germline mutations in these genes markedly increase the risk for hereditary breast and ovarian cancers [12]. TERT (Telomerase Reverse Transcriptase), which maintains telomere length, is commonly overexpressed in breast tumors and contributes to oncogenic transformation and cellular immortality [13]. Variants in FGFR2 (Fibroblast Growth Factor Receptor 2) have been identified through genome‐wide association studies and are associated with an elevated risk of estrogen receptor‐positive BC [14]. CHD1 (Chromodomain Helicase DNA Binding Protein 1), a chromatin remodeling factor, has also been implicated in BC biology, particularly in contexts involving PTEN deficiency, affecting gene expression and tumor growth [15].

## 2. Materials and Methods

### 2.1. Study Cohort and Tissue Sample Collection

This retrospective study included 125 formalin‐fixed, paraffin‐embedded (FFPE) tissue samples diagnosed histopathologically as BC, collected between 2023 and 2024. Patients who had undergone radiation, chemotherapy, or oncologic surgery were excluded. The FFPE tissue sections were obtained through colposcopy‐guided biopsy at King Fahad Medical City in Riyadh, Saudi Arabia. The control group included 25 nonmalignant breast tissue samples. Clinicopathological data for all patients were retrospectively gathered from the computerized clinical information system.

### 2.2. MMTV Detection by Quantitative Polymerase Chain Reaction (qPCR)

FFPE tissue samples underwent viral DNA extraction using the AllPrep DNA/RNA FFPE Kit (Qiagen, Valencia, CA, United States), following the protocol provided by the manufacturer to ensure optimal yield and purity. The MMTV (VR 732) positive control DNA was isolated using the Viral Nucleic Acid Extraction Kit II (VR300) from Geneaid (New Taipei City, Taiwan), following the supplier′s instructions. Detection of MMTV was performed using a set of specific primers targeting different regions of the MMTV genome (Table S1). To assess the quality of the extracted nucleic acids and ensure the absence of PCR inhibitors, amplification of two internal control genes, GAPDH and *β*‐globin, was carried out. Quantitative PCR assays were prepared using a SYBR Green‐based master mix containing ROX as a passive reference dye. Each 20‐*μ*L reaction comprised 10 *μ*L of 2× RT^2^ SYBR Green Master Mix, 1 *μ*L of forward and reverse primers, 2 *μ*L of extracted DNA, and 6 *μ*L of RNase‐free water. Thermal cycling was initiated with a denaturation step at 95°C for 10 min, followed by 40 amplification cycles consisting of 15 s at 95°C for denaturation, 45 s at 57°C for primer annealing, and 45 s at 72°C for extension. Fluorescence data were collected during the extension step. The protocol concluded with a final elongation at 72°C for 5 min. A melt curve analysis was performed postamplification by increasing the temperature incrementally from 60°C to 95°C at a rate of 10°C per step to assess product specificity. The MMTV (VR 732) positive control was sourced from the American Type Culture Collection (ATCC). Each run included both positive and negative controls, and a sample was deemed MMTV‐positive if the cycle threshold (Ct) value was less than 35 and the melting peak was within ±0.5°C of the positive control.

### 2.3. Gene Expression Analysis by qPCR

Expression levels of TP53, BRCA1, BRCA2, TERT, FGFR2, and CHD1 transcripts were quantified using real‐time PCR with gene‐specific primer sets (see Table S1). Total RNA was extracted from FFPE tissues using the AllPrep DNA/RNA FFPE Kit (Qiagen, Valencia, CA, United States), adhering to the protocol supplied by the manufacturer. First‐strand complementary DNA (cDNA) synthesis was performed using the QuantiTect Reverse Transcription Kit (Qiagen) according to the manufacturer′s guidelines. Quantitative gene expression analysis was then carried out using the QuantiTect SYBR Green PCR Kit and QuantiTect Primer Assays (Qiagen). Relative gene expression was determined using the 2^−*ΔΔ*Ct method, with GAPDH serving as the endogenous reference for normalization.

### 2.4. Statistical Analysis

Categorical variables were expressed as counts and percentages and compared using chi‐square or Fisher′s exact tests. Continuous data were analyzed with *t*‐tests or ANOVA if normally distributed and Mann–Whitney *U* or Kruskal–Wallis tests if not. Recurrence‐free survival (RFS) and overall survival (OS) were estimated using the Kaplan–Meier method and compared with the log‐rank test. A *p* value < 0.05 was considered significant. Analyses were performed using SPSS v25.0 (IBM Corp., Armonk, NY, United States).

## 3. Results

### 3.1. Patients′ Characteristics

The study included 25 patients with benign breast lesions and 125 patients with BC. In the benign group, the mean age was 39.8 ± 13.63 years, with 44% of patients below the mean age. The mean BMI was 28.34 ± 6.83 kg/m^2^, with 64% of patients having BMI below the mean. Among BC patients, the mean age was 54.81 ± 10.92 years, with an almost equal distribution of patients above and below the mean age (50.4% vs. 49.6%). The median BMI was 32 kg/m^2^ (IQR: 29–35), with 58.4% of patients below the median. The predominant histological type was invasive ductal carcinoma, not otherwise specified (IDC‐NOS) (93.7%), followed by invasive lobular carcinoma (ILC) (4%). Regarding tumor staging, most patients presented with T2 (45.6%), N1 (48.8%), and M0 (96.8%) disease. The majority of patients (73.6%) received combined surgery and chemoradiotherapy. During follow‐up, 9.6% of patients experienced disease recurrence, and the mortality rate was 4% (Table 1).

**Table 1:** Demographic and clinical characteristics of patients.

Benign breast lesions (*n* = 25)		
Age (years)	Mean ± SD	39.8 ± 13.63
	≤ Mean age, *n* (%)	11 (44%)
	> Mean age, *n* (%)	14 (56%)
BMI (kg/m^2^)	Mean ± SD	28.34 ± 6.83
	≤ Mean BMI, *n* (%)	16 (64)
	> Mean BMI, *n* (%)	09 (36)
Breast cancer (*n* = 125)		
Age (years)	Mean ± SD	54.81 ± 10.92
	≤ Mean age, *n* (%)	63 (50.40)
	> Mean age, *n* (%)	62 (49.60)
Body mass index (kg/m^2^)	Mean ± SD	32 (29–35)
	≤ Median BMI, *n* (%)	73 (58.40)
	> Median BMI, *n* (%)	52 (41.60)
Tumor histology	Invasive ductal carcinoma, not otherwise specified (IDC‐NOS), *n* (%)	117 (93.6)
	Invasive lobular carcinoma (ILC), *n* (%)	5 (4.0)
	Invasive papillary carcinoma (IPC), *n* (10%)	1 (0.8)
	Ductal carcinoma in situ (DCIS), *n* (%)	2 (1.6)
TNM staging system	T1, *n* (%)	35 (28.00)
	T2, *n* (%)	57 (45.60)
	T3, *n* (%)	23 (18.40)
	T4, *n* (%)	10 (8.00)
Regional lymph nodes (N) category	N0, *n* (%)	48 (38.40)
	N1, *n* (%)	61 (48.80)
	N2, *n* (%)	10 (8)
	N3, *n* (%)	6 (4.80)

Distant metastasis (M) category	M0, *n* (%)	121 (96.80)
	M1, *n* (%)	4 (3.20)

Treatment strategies	Surgery only, *n* (%)	5 (4)
	Surgery + chemotherapy, *n* (%)	22 (17.60)
	Surgery + radiotherapy, *n* (%)	6 (4.80)
	Surgery + chemotherapy + radiotherapy, *n* (%)	92 (73.60)
Recurrence	Yes, *n* (%)	12 (9.60)
	No, *n* (%)	113 (90.40)
Recurrence‐free survival (months)	Yes, *n* (%)	27 (22–33) 112 (89.60)
	No, *n* (%)	13 (10.40)
Overall survival (months)	Alive, *n* (%)	29 (25–35) 120 (96.00)
	Deceased, *n* (%)	5 (4.00)

### 3.2. Quantification of MMTV Using Real‐Time PCR

Twenty‐five patients with benign breast lesions and 125 patients with BC were screened for the presence of MMTV. Despite using the positive control MMTV (VR‐732) from the ATCC and including both positive and negative controls in each PCR run, the results were negative for MMTV. Reactions with a cycle threshold (Ct) of less than 35 and a melting peak difference of less than 0.5°C from the positive control were considered positive, but none of the samples met these criteria.

### 3.3. Expression of p53, BRCA1, BRCA2, TERT, FGFR2, and CHD1 Genes

Analysis of gene expression levels revealed distinct patterns between noncancerous breast tissue and BC tissue for most of the studied genes. The expression levels were quantified as log2 fold change, with significant differences observed in five out of the six analyzed genes. P53 expression showed a significant difference between the tissue types (*p* < 0.001), with BC tissue showing slightly higher expression levels. The distribution of p53 expression was more compact in noncancerous tissue, while BC tissue showed several outliers at higher expression levels (Figure 1a). BRCA1 expression showed significantly lower levels in BC tissue compared to noncancerous breast tissue (*p* = 0.001). The box plot revealed a narrower distribution of BRCA1 expression in BC tissue, suggesting more consistent downregulation in cancerous cells (Figure 1b). BRCA2 demonstrated a more pronounced downregulation in BC tissue (*p* < 0.001), with the median expression substantially lower compared to noncancerous tissue. Several outliers were observed in the BC tissue group, indicating some heterogeneity in BRCA2 expression among cancer patients (Figure 1c). TERT expression was significantly lower in BC tissue compared to noncancerous tissue (*p* < 0.001). The expression pattern in BC tissue showed a narrow distribution with few outliers, whereas noncancerous tissue demonstrated a wider range of expression levels (Figure 1d). FGFR2 was the only gene that showed no significant difference in expression between cancerous and noncancerous tissues (*p* = 0.300). Both tissue types exhibited similar expression patterns with some outliers in both groups, indicating that FGFR2 expression may not be consistently altered in BC (Figure 1e). CHD1 expression was significantly reduced in BC tissue (*p* < 0.001), showing a marked difference between the two tissue types. The expression in BC tissue showed a compact distribution with minimal outliers, suggesting consistent downregulation of this gene in BC (Figure 1f).

Figure 1Relative expression profiles of p53, BRCA1, BRCA2, TERT, FGFR2, and CHD1 genes in BC tissue samples versus noncancerous breast tissue samples. Data are presented as median values with interquartile ranges (IQR), defined as the span from the 25th to the 75th percentile.

**Figure:**
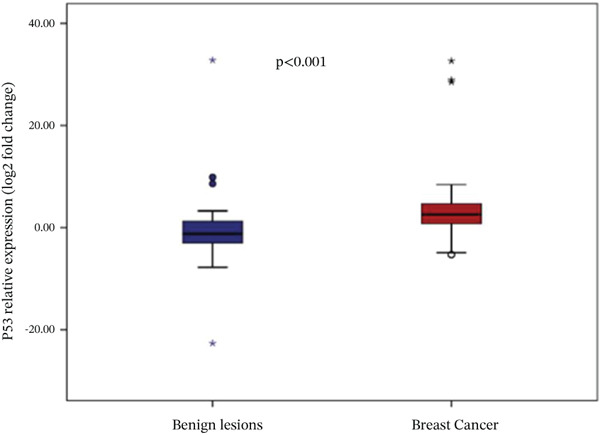
(a)

**Figure:**
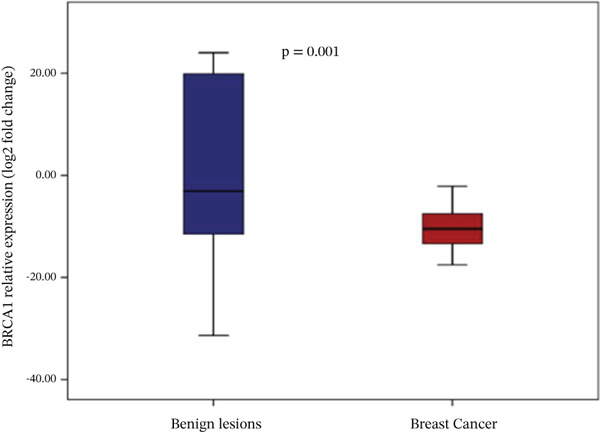
(b)

**Figure:**
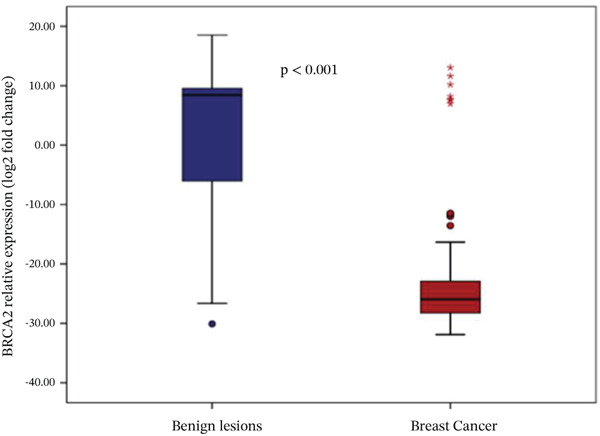
(c)

**Figure:**
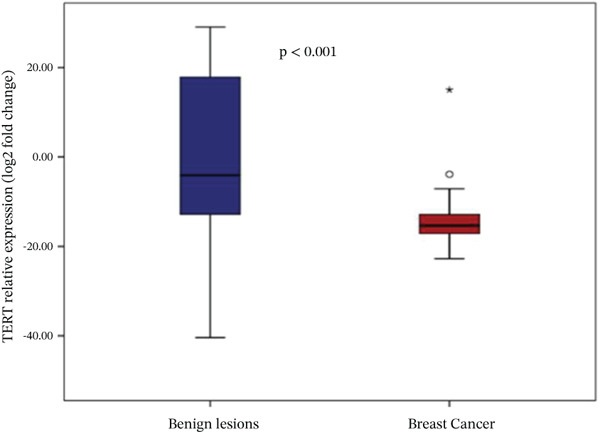
(d)

**Figure:**
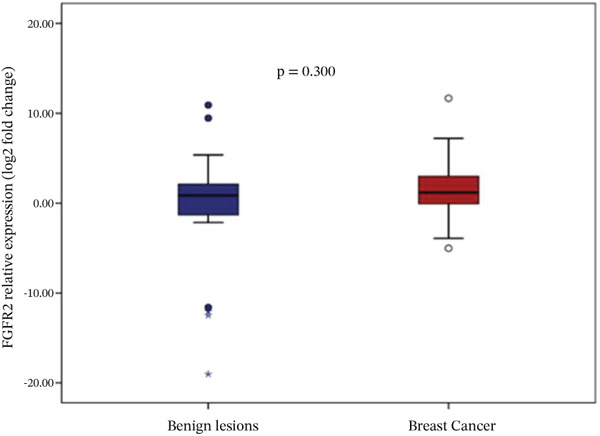
(e)

**Figure:**
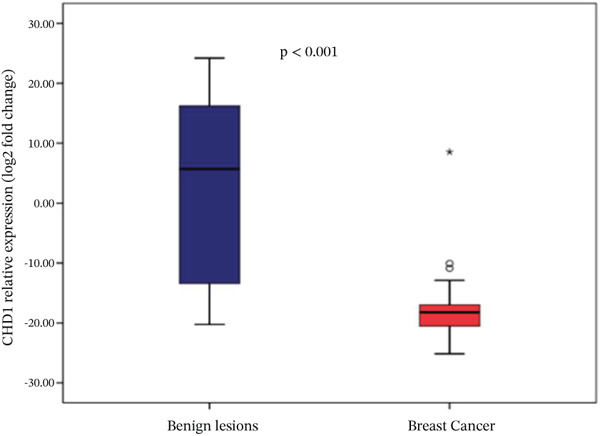
(f)

### 3.4. Association of High and Low Genes Expressions With RFS and OS

Subsequent analysis to identify independent prognostic factors for RFS and OS in BC patients showed that some gene expression levels were significant prognostic factors. Patients with high p53 expression demonstrated longer RFS (28.5 vs. 24 months, *p* = 0.004) and OS (31 vs. 28 months, *p* = 0.042) compared to those with low expression. Similarly, BRCA1 expression analysis revealed that patients with high expression levels had significantly longer RFS (32 vs. 24 months, *p* < 0.001) and OS (34 vs. 26 months, *p* < 0.001) compared to those with low expression. For BRCA2, TERT, FGFR2, and CHD1 gene expression, no statistically significant associations were observed with RFS or OS (all *p* > 0.05) High p53 and BRCA1 expressions emerged as a significant prognostic factor for RFS and OS in the entire series of BC patients, indicating their strong predictive value for survival outcomes in BC patients (Table 2).

**Table 2:** Prognostic significance of recurrence‐free survival (RFS) and overall survival (OS) in the complete cohort of breast cancer (BC) patients.

Gene expression	Survival type	Breast cancer (*n* = 125)		*p* value
		Low (*n* = 63)	High (*n* = 62)	
p53	RFS (months)	24 (21–29)	28.5 (26–34)	0.004∗
	Yes, *n* (%)	54 (85.7)	58 (93.5)	
	No, *n* (%)	9 (14.3)	4 (6.5)	
	OS (months)	28 (24–34)	31 (27–35)	0.042∗
	Alive, *n* (%)	60 (95.2)	60 (96.8)	
	Dead, *n* (%)	3 (4.8)	2 (3.2)	
BRCA1	RFS (months)	24 (21–28)	32 (27–35)	< 0.001∗
	Yes, *n* (%)	57 (90.5)	55 (88.7)	
	No, *n* (%)	6 (9.5)	7 (11.3)	
	OS (months)	26 (23–30)	34 (28–36)	< 0.001∗
	Alive, *n* (%)	60 (95.2)	60 (96.8)	
	Dead, *n* (%)	3 (4.8)	2 (3.2)	
BRCA2	RFS (months)	27 (24–32)	27 (21–34)	0.630
	Yes, *n* (%)	56 (88.9)	56 (90.3)	
	No, *n* (%)	7 (11.1)	6 (9.7)	
	OS (months)	29 (25–35)	28 (24–35)	0.870
	Alive, *n* (%)	61 (96.8)	59 (95.2)	
	Dead, *n* (%)	2 (3.2)	3 (4.8)	
TERT	RFS (months)	27 (22–32)	27 (22–34)	0.420
	Yes, *n* (%)	56 (88.9)	56 (90.3)	
	No, *n* (%)	7 (11.1)	6 (9.7)	
	OS (months)	28 (24–34)	31 (26–35)	0.103
	Alive, *n* (%)	59 (93.7)	61 (98.4)	
	Dead, *n* (%)	4 (6.3)	1 (1.6)	
FGFR2	RFS (months)	27 (22–32)	28 (22–34)	0.119
	Yes, *n* (%)	56 (88.9)	56 (90.3)	
	No, *n* (%)	7 (11.1)	6 (9.7)	
	OS (months)	28 (24–34)	30.5 (26–35)	0.155
	Alive, *n* (%)	61 (96.8)	59 (95.2)	
	Dead, *n* (%)	2 (3.2)	3 (4.8)	
CHD1	RFS (months)	27 (23–32)	27 (22–33.25)	0.968
	Yes, *n* (%)	58 (92.1)	54 (87.1)	
	No, *n* (%)	5 (7.9)	8 (12.9)	
	OS (months)	28 (24–35)	29.5 (25–35)	0.688
	Alive, *n* (%)	61 (96.8)	59 (95.2)	
	Dead, *n* (%)	2 (3.2)	3 (4.8)	

∗Indicates a statistically significant difference.

### 3.5. Association Between Gene Expression Profiles and RFS and OS in BC Patients

**Figure 2:**
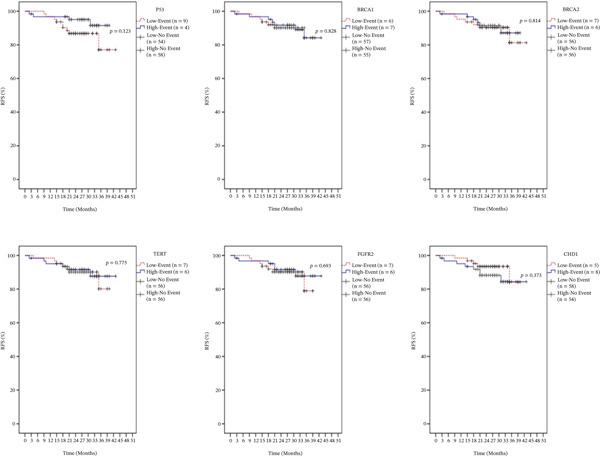
Analysis of recurrence‐free survival across the entire breast cancer patient cohort based on high versus low expression of p53, BRCA1, BRCA2, TERT, FGFR2, and CHD1.

Kaplan–Meier survival analyses were conducted to evaluate RFS based on dichotomized gene expression levels (low vs. high) for p53, BRCA1, BRCA2, TERT, FGFR2, and CHD1. Patient distribution according to expression level and event status is illustrated in Figure 2. Among the analyzed genes, p53 showed the lowest *p* value (*p* = 0.123), though not statistically significant. Notably, survival curves remained relatively stable for both expression groups up to 43 months, with a trend suggesting improved RFS in the low‐expression group. BRCA1 expression levels did not significantly affect RFS (*p* = 0.828), and survival trajectories for both high and low expression groups remained comparable throughout the 45‐month follow‐up period. Similarly, BRCA2 expression exhibited no significant relationship with RFS (*p* = 0.814), with survival curves closely resembling those of BRCA1, indicating substantial overlap between the groups. TERT expression analysis yielded no statistically significant association with RFS (*p* = 0.775). Likewise, no significant differences in RFS were observed between FGFR2 expression groups (*p* = 0.693). Analysis of CHD1 expression also revealed no significant prognostic impact (*p* = 0.373), with both expression groups showing parallel survival trends throughout the study period (Figure 2).

**Figure 3:**
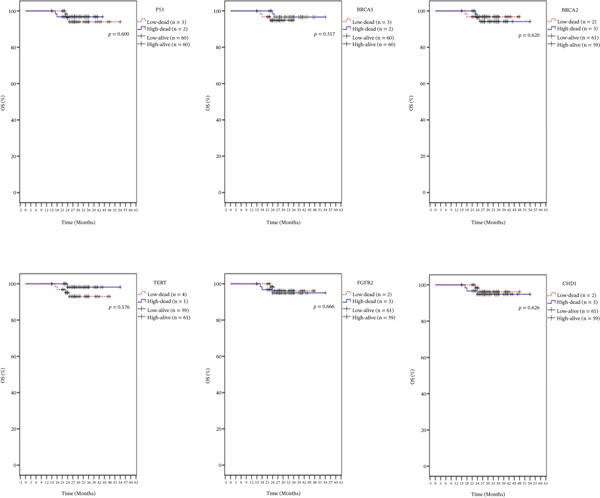
Overall survival (OS) of the entire series of BC patients stratified by p53, BRCA1, BRCA2, TERT, FGFR2, and CHD1 genes expression status (low/high).

Kaplan–Meier survival analyses were performed to explore the association between gene expression levels and OS for the six genes under investigation. The analysis of p53 expression revealed no statistically significant effect on OS (*p* = 0.600). There were three deaths among patients with low expression and two in the high‐expression group, while 60 patients in each category survived. Although the survival curves showed limited separation, the low‐expression group exhibited a slightly longer median survival of 54 months (Figure 3). Similarly, BRCA1 expression was not significantly associated with OS (*p* = 0.517), with most individuals in both expression categories surviving throughout the observation period. BRCA2 expression also showed no meaningful relationship with OS (*p* = 0.620). FGFR2 expression was not significantly correlated with OS (*p* = 0.666), and survival trends for CHD1 remained largely parallel between expression groups during the follow‐up period (Figure 3).

## 4. Discussion

All 150 samples analyzed by quantitative PCR (qPCR), including those from patients with benign breast lesions and BC, tested negative for MMTV. These findings do not support an association between MMTV and the development of BC in the studied cohort. However, the presence of MMTV in human BC remains controversial, with several studies failing to consistently detect viral sequences in human breast tissue [16, 17]. Despite the association of MMTV with mammary tumors in animal models, epidemiological data and molecular studies in humans have yielded mixed results, leaving the exact role of MMTV in human BC uncertain [18, 19]. Several studies, including one from Saudi Arabia, have reported inconsistent detection of MMTV‐like sequences in 8.7% of BC tissue samples, reflecting a relatively low positivity rate and adding to the global inconsistency in detecting viral sequences in breast tissue [20]. While the investigation into MMTV′s role in human BC continues, current evidence does not support its widespread involvement in the disease.

This study investigated the expression profiles and prognostic relevance of key genes involved in tumor suppression, telomere maintenance, DNA damage response, and DNA repair in BC. The analysis revealed significant differential expression in five of the six studied genes between BC and noncancerous breast tissue. The findings suggest a complex pattern of gene expression changes in BC, with significant downregulation of BRCA1, BRCA2, CHD1, and TERT, upregulation of p53, and unchanged levels of FGFR2. The significant upregulation of p53 in BC tissue is in line with the well‐documented role of P53 mutations and the accumulation of dysfunctional p53 protein in a large proportion of BC cases [21]. The downregulation of BRCA1, BRCA2 [22], TERT [23], and CHD1 [24] in BC tissue also aligns with prior findings. Their reduced expression in sporadic BC has been associated with genomic instability and tumor suppression. FGFR2 was the only gene without a significant expression difference, indicating that FGFR2 mRNA levels may not be of prognostic value in BC. Although FGFR2 expression at the transcript level may appear stable, functional impacts may still exist at the regulatory, isoform, or protein activity level [25]. These significant gene expression differences should be interpreted in the context of their prognostic utility for RFS and OS. The observed discrepancies might reflect the heterogeneous nature of BC or indicate that the prognostic value of these genes is context dependent and potentially modulated by additional molecular or clinical factors.

The prognostic significance of six tumor‐associated genes (P53, BRCA1, BRCA2, TERT, FGFR2, and CHD1) expression for RFS and OS in BC patients was evaluated over 63 months. Among the investigated genes, only p53 and BRCA1 expression levels emerged as significant prognostic factors for both RFS and OS in the entire cohort, indicating their strong predictive value for survival outcomes in BC. Patients with high p53 expression had longer RFS (28.5 vs. 24 months) and OS (31 vs. 28 months), while those with high BRCA1 expression showed even greater benefit in both RFS (32 vs. 24 months) and OS (34 vs. 26 months). This suggests that higher expression of p53 and BRCA1 may be indicative of a less aggressive tumor phenotype and better prognosis in BC patients. It also implies that these genes could serve as potential prognostic biomarkers, helping to stratify patients based on their risk and guide treatment decisions. Overall, this highlights the complexity of BC biology and the need for integrated molecular profiling to fully understand prognostic markers.

Kaplan–Meier survival analyses were performed to evaluate the prognostic significance of p53, BRCA1, BRCA2, TERT, FGFR2, and CHD1 gene expression in relation to OS and RFS. Although none of the six genes studied showed a statistically significant impact on OS or RFS, only p53 gene displayed a slightly different pattern of events concerning RFS and OS when comparing the low‐expression group to the high‐expression group. Therefore, the variation observed in RFS and OS is likely due to factors beyond gene expression. While these genes are mechanistically relevant to tumor biology, their limited prognostic value in this study emphasizes the need for comprehensive future research with larger cohorts.

In this study, p53 expression displayed a trend favoring low expression for better survival, reflecting the underlying biology of p53 regulation and mutation. In BC, high p53 expression is often associated with missense mutations, which result in the accumulation of a nonfunctional, mutant form of p53 that lacks tumor‐suppressive activity and may even gain oncogenic functions [21, 26]. The mutation of the TP53 gene is most important, as mutations can impair p53′s ability to regulate the cell cycle and induce apoptosis [27]. The interactions of the tumor microenvironment (i.e., immune cells and stromal components) with p53 also compromise p53 activity [28]. Concurrent genetic aberrations, such as mutations in oncogenes or tumor suppressor genes, could also influence the role of p53 in tumor development [29]. The apoptotic activity of p53 can be modulated by treatments such as chemotherapy or radiotherapy, which can contribute to survival and the course of the disease [30]. Finally, epigenetic modifications can alter p53 expression and function without changing the DNA sequence, further complicating its role in survival.

This study has some limitations. Firstly, the relatively small sample size, particularly in the benign lesion group, may have limited the statistical power to detect significant differences or associations. Secondly, the follow‐up period was relatively short, which may have influenced the ability to observe long‐term survival outcomes. Thirdly, the analysis was limited to gene expression at the mRNA level, without complementary protein‐level data, which is particularly relevant for genes like p53 where post‐transcriptional regulation plays a critical role. For future perspectives, it is essential to consider the mutation status of key genes such as TP53, BRCA1, and BRCA2, as this provides a more accurate understanding of their functional impact in BC. Relying solely on gene expression levels can be misleading, since wild‐type and mutant forms often exhibit opposing biological and prognostic behaviors. Integrating mutation analysis alongside expression profiling would offer a more comprehensive insight into their roles in tumor progression and patient outcomes.

## 5. Conclusions

This study provides exploratory insights into the molecular features of BC in the analyzed cohort. No evidence of MMTV was detected in any of the examined breast tissue samples. We further evaluated the expression patterns of six BC‐related genes involved in tumor suppression, telomere maintenance, and DNA damage response. Differential expression between cancerous and noncancerous tissues was observed for BRCA1, BRCA2, CHD1, TERT, and p53, while FGFR2 expression remained unchanged. Survival analyses indicated that BRCA1 and p53 expression levels were associated with recurrence‐free and OS in univariate analyses. However, given the limited sample size and number of events, these findings should be interpreted with caution, as multivariate adjustment for potential confounders was not feasible. The observed associations suggest a potential prognostic relevance of BRCA1 and p53 expression, but they do not establish independent prognostic value.

Overall, while the results support previously reported trends and contribute additional data, larger studies with longer follow‐up, multivariate modeling, and integration of additional molecular and clinical variables will be necessary to validate these observations and clarify their clinical utility.

## Author Contributions

Conceptualization, S.A. (Saad Alamri), M.A.M.A. (Mohamed A.M. Ali), M.A. (Maaweya Awadalla), and B.A. (Bandar Alosaimi); methodology, S.A. (Saad Alamri), M.A. (Maaweya Awadalla), G.A. (Ghaida Al‐hazzaa), and R.H. (Rahaf Henawi); software, S.A. (Saad Alamri) and M.A. (Maaweya Awadalla); formal analysis, A.S.A.H. (Alyaa S. Abdel Halim), S.A. (Saad Alamri), and M.A.M.A. (Mohamed A.M. Ali); investigation, S.A. (Saad Alamri) and M.A. (Maaweya Awadalla); data curation, Z.A. (Zahra Alkhunaizy), S.A. (Soha Alzorgi), and N.A. (Nouf Alqahtani); writing original draft preparation, S.A. (Saad Alamri) and M.A.M.A. (Mohamed A.M. Ali); writing, review, and editing, B.A. (Bandar Alosaimi) and M.A. (Maaweya Awadalla); visualization, B.A. (Bandar Alosaimi); funding acquisition, S.A. (Saad Alamri).

## Funding

This study was supported by King Fahad Medical City, 10.13039/501100011680, Grant Number 022‐035.

## Disclosure

All authors have read and agreed to the published version of the manuscript.

## Ethics Statement

The research adhered to the ethical principles established in the Declaration of Helsinki. This retrospective study, along with the utilization of clinical data and tissue samples, received approval from the institutional review board (IRB) committee of King Fahad Medical City (IRB Log No. 22‐533, Riyadh, Saudi Arabia) in accordance with national and international guidelines governing the appropriate use of clinical materials.

## Consent

Informed consent was not acquired for the utilization of retrospective tissue samples from the study′s subjects. The patients in this study cannot be recognized through their clinical information, and informed consent for gene expression analysis was unnecessary due to the retrospective design of the study.

## Conflicts of Interest

The authors declare no conflicts of interest.

## Supporting information


**Supporting Information** Additional supporting information can be found online in the Supporting Information section. Table S1: List of primers that used host gene expression.

## Data Availability

The data sets generated or analyzed during the current study are available from the corresponding author upon reasonable request.
