# Supplementary material for: MMTV Virus Detection, Survival Analysis, and Prognostic Relevance of Six Tumor Genes in Patients With Breast Cancer
Source: Int J Breast Cancer. 2026 Apr 12;2026:3310843. doi: 10.1155/ijbc/3310843 (PMC13071179; doi:10.1155/ijbc/3310843)
Supplement: Supplementary file 1 — Supporting Information Additional supporting information can be found online in the Supporting Information section. Table S1: List of primers that used host gene expression. [file IJBC-2026-3310843-s001.docx]

**Supplementary Tables:**

**Supplementary Table 1: List of Premiers Used for Host Gene Expression**

| BRCA 1 | Forward 5′- CTGAAGACTGCTCAGGGCTATC-3′ |
| --- | --- |
| BRCA 1 | Reverse 5′- AGGGTAGCTGTTAGAAGGCTGG -3′ |
| BRCA 2 | Forward 5′-GATCTTTAACTGTTCTGGGTCACA-3′ |
| BRCA 2 | Reverse 5′-CCCAGCATGACACAATTAATGA-3′ |
| CDH1 | Forward 5’- GCCTCCTGAAAAGAGAGTGGAAG -3’ |
| CDH1 | Reverse 5’- TGGCAGTGTCTCTCCAAATCCG -3’ |
| FGFR2 | Forward 5’ - GTGCCGAATGAAGAACACGACC-3’ |
| FGFR2 | Reverse 5’- GGCGTGTTGTTATCCTCACCAG -3’ |
| TERT | Forward 5’- GCCGATTGTGAACATGGACTACG-3’ |
| TERT | Reverse 5’- GCTCGTAGTTGAGCACGCTGAA -3’ |
| TP53 | Forward 5’- CCTCAGCATCTTATCCGAGTGG -3’ |
| TP53 | Reverse 5’- TGGATGGTGGTACAGTCAGAGC -3’ |

**Supplementary Table 2: List of Premiers Used for MMTV detection**

| MMTV 1 | MMTV1-F 5’-ATGGGTAGAACCTACWTGGTTCTG-3’ |
| --- | --- |
| MMTV 2 | MMTV1-R 5’-ATAAGGRTAAGTAACACAGGCAGA-3′ |
| MMTV 3 | MMTV2-F 5’-GCTCTAGTTCCCCATACAGA-3′ |
| MMTV 4 | MMTV1-R 5’-GCAGATGTAGGAATCATCTCATG-3′ |
